# New Appliances and Things Medical

**Published:** 1901-01-05

**Authors:** 


### NEW APPLIANCES AND THINGS MEDICAL.

[We shall be glad to receive, at our Office, 28 & 29 Southampton Street, Strand, London, W.C., from the manufacturers, specimens of all new preparations and appliances which may be brought out from time to time.]

### PRICE'S ASEPTIC AND ANTISEPTIC SOAPS.

(PRICE'S PATENT CANDLE COMPANY, LIMITED, BELMONT WORKS, BATTERSEA, LONDON, S.W.)

WE have examined three varieties of medical soap manufactured by the above company. The first is called "zincoleate," and contains the oxide and oleate of zinc. The combination of these dermatological medicaments with a soap basis supplies a useful medium for the cleansing of skin, which is the seat of eczematous or other inflammatory lesions. The mercoleate variety is a soap which contains 5 per cent. freshly precipitated oleate of mercury. As an antiseptic and dermatological soap it should be invaluable in all cases in which mercury is not contraindicated. The third variety, an aseptic soap, is a particularly useful and non-poisonous preparation: the medicinal contents are somewhat of the same nature as those of that invaluable antiseptic Listerine, for they consist of thymol, menthol, eucalyptus, wintergreen, sodium benzoate, &c. Such a combination of non-poisonous antiseptics should render this soap invaluable for nurses, especially on occasions when a powerful antiseptic is unnecessary.

### KUMOFF AND NETTWAR.

(THE WALL PAPER CLEANING SYNDICATE, LIMITED, 22 GLASSHOUSE STREET, LONDON, W.)

These two preparations are employed by the above company for cleaning walls, painted and varnished surfaces, &c. Kumoff is a plastic substance somewhat of the consistence of putty; Nettwar is a liquid. Whether the solid or liquid substance should be used for the cleaning of any particular object depends upon the nature of the surface. If porous and absorbent and likely to be damaged by a fluid, the choice should rest with Kumoff; otherwise Nettwar is the simpler and easier to apply. With each of these cleansers a certain proportion of carbolic acid can be incorporated, an addition which may be considered desirable for the cleaning or disinfection of hospital wards, or public rooms, lavatories, workhouses, &c. The method of use is quite simple, and the directions supplied with the preparations are such as to render the application perfectly easy in the hands of any intelligent persons. Both Kumoff and Nettwar will enable the ordinary domestic staff in any private establishment to perform with complete success the cleaning work which is generally left to a professional.

